# Advances in Hyperspectral Imaging Technology for Grain Quality and Safety Detection: A Review

**DOI:** 10.3390/foods14172977

**Published:** 2025-08-26

**Authors:** Yuting Liang, Zhihua Li, Jiyong Shi, Ning Zhang, Zhou Qin, Liuzi Du, Xiaodong Zhai, Tingting Shen, Roujia Zhang, Xiaobo Zou, Xiaowei Huang

**Affiliations:** 1 Agricultural Product Processing and Storage Lab, School of Food and Biological Engineering, Jiangsu University, Zhenjiang 212013, China; 15293170917@163.com (Y.L.);; 2 College of Food Science and Engineering, Nanjing University of Finance and Economics/Collaborative Innovation Center for Modern Grain Circulation and Safety, Nanjing 210023, China

**Keywords:** hyperspectral imaging, nondestructive testing, spectral analysis, grain safety, adulteration detection, disease detection, geographical origin tracing

## Abstract

This review provides an overview of recent advancements in hyperspectral imaging (HSI) technology for grain quality and safety detection, focusing on its impact on global food security and economic stability. Traditional methods for grain quality assessment are labor-intensive, time-consuming, and destructive, whereas HSI offers a non-destructive, efficient, and rapid alternative by integrating spatial and spectral data. Over the past five years, HSI has made significant strides in several key areas, including disease detection, quality assessment, physicochemical property analysis, pesticide residue identification, and geographic origin determination. Despite its potential, challenges such as high costs, complex data processing, and the lack of standardized models limit its widespread adoption. This review highlights these advancements, identifies current limitations, and discusses the future implications of HSI in enhancing food safety, traceability, and sustainability in the grain industry.

## 1. Introduction

Grains are foundational to global nutrition and economic stability, serving as the primary source of carbohydrates, proteins, and micronutrients for billions of people while forming diverse food products, from staple foods like rice and wheat to processed items such as noodles, porridge, bread and pastries [1,2]. In agrarian economies like China, grains are critical to both food security and economic growth [3,4]. According to the Food and Agriculture Organization (FAO) Statistical Yearbook 2024, global production of major crops totaled 9.9 billion tons, with maize, wheat, and rice comprising 91% of total cereal output. However, climate volatility, soil degradation, and pest resistance threaten yield stability [5,6], increasing the need for efficient, nondestructive quality assessment methods to ensure food security [7].

Grain quality assessment is essential for evaluating both market value and suitability for consumption. Conventional physical grading and colony detection are time-consuming, limited to surface evaluations, and unable to resolve internal properties such as chemical composition and susceptibility to diseases or contaminants [8,9]. Techniques like high performance liquid chromatography (HPLC), gas chromatography (GC), and mass spectrometry (MS) exhibit high sensitivity and specificity in detecting pesticide residues, mycotoxins, and chemical compositions in grains [10,11]. However, these methods are often destructive, subjective, and require complex sample pretreatment, making them time-consuming [12,13]. In contrast, spectroscopy provides a promising nondestructive alternative, enabling faster, more comprehensive analysis of grain properties without damaging the samples [14,15,16].

Among common spectral techniques, near-infrared spectroscopy enables rapid detection of chemical substances such as moisture and protein in grains by leveraging molecular overtone vibrations [1]. However, its application in structural analysis is limited, as it provides only spectral data without spatial details. Fluorescence spectroscopy allows for the detection of mycotoxins in cereals by inducing characteristic fluorescence in some mycotoxins under specific wavelength excitation [17]. But its signal is sensitive to environmental factors, reducing detection accuracy. Hyperspectral imaging (HSI) combines the spatial resolution of traditional imaging with spectral data from individual pixels across a broad wavelength range, enabling the extraction of both spatial and chemical information from a single scan [18]. It has emerged as a powerful nondestructive tool for assessing agricultural product quality [19]. This technique enables rapid assessment by establishing regression models between hyperspectral imaging data of collected grains and chemical parameters (e.g., protein, moisture, fat, and carbohydrate content).

**Figure 1:**
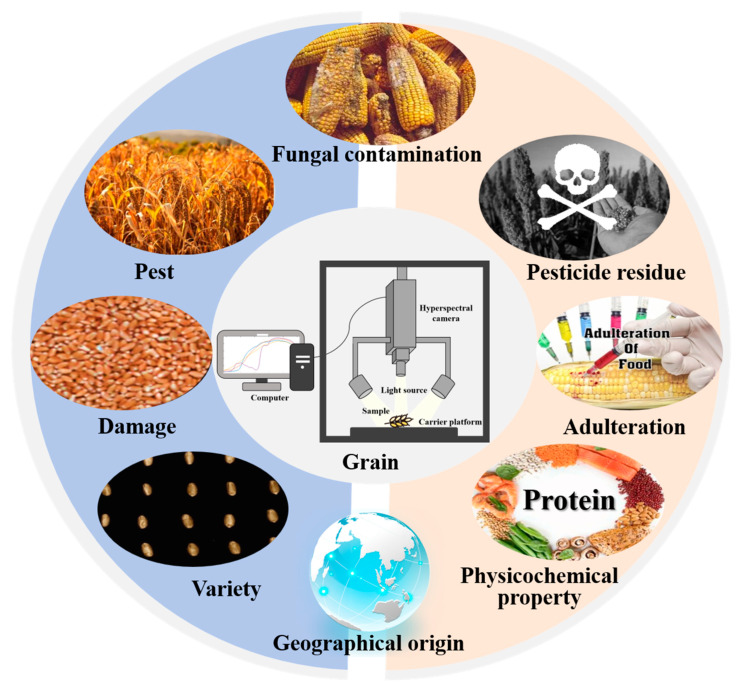
Core applications of hyperspectral imaging in grain quality and safety detection: (1) fungal contamination detection, (2) pesticide residue detection, (3) physicochemical property analysis, (4) variety identification, (5) damage detection, (6) pest detection, (7) geographical origin determination, and (8) adulteration detection.

Computational analysis of pixel spectra quantifies chemical indicators and generates detailed spatial distribution maps [2,20]. As shown in Figure 1, this dual capability facilitates comprehensive grain quality analysis, including the detection and quantification of fungal contamination, pesticide residues, physicochemical properties, variety, damage, and pest detection [21,22].

**Figure 2:**
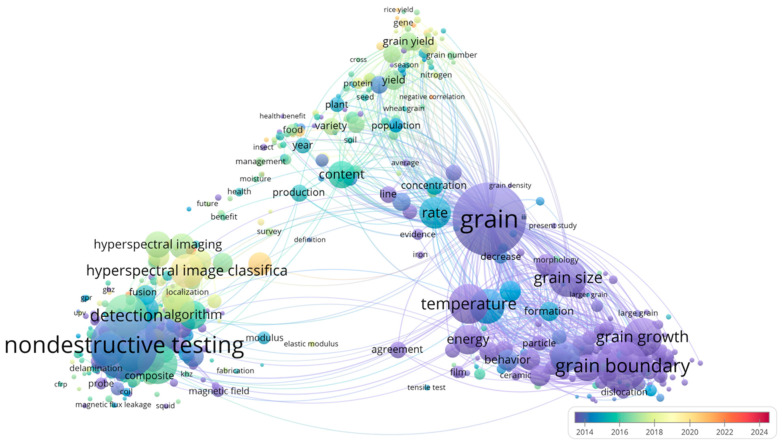
VOS viewer-based network map of hotspots and trends co-occurring in hyperspectral imaging research: agriculture and nondestructive testing.

Figure 2 illustrates the key research areas and trends in hyperspectral imaging based on the core themes of “hyperspectral imaging”, “grain”, and “nondestructive testing”. The research is grouped into three primary domains: agriculture, nondestructive testing, and data processing. In agriculture, the focus is on yield, quality (e.g., “protein”, “content”), and growth monitoring, underscoring its relevance in precision farming. Nondestructive testing emphasizes composite material characterization, surface defect analysis, and algorithm development (e.g., “detection algorithm”). Temporal trends reveal a shift from early studies on physical property characterization to recent advances in machine learning-based classification (“classification”). Hyperspectral imaging is evolving from traditional approaches toward more sophisticated, intelligent applications, particularly in deep learning and feature optimization.

This review summarizes advancements in hyperspectral imaging technology over the past five years, focusing on its principles, system components, and progress in grain detection. We highlight key applications in disease detection, quality assessment, physicochemical property analysis, pesticide residue detection, and geographical origin determination. Prior to these advancements, hyperspectral imaging faced several limitations, including high costs, low spatial resolution, and complex data processing [23,24,25,26]. We also discuss current challenges and explore promising future directions for research and technological development.

## 2. Hyperspectral Imaging Technology

### 2.1. Components of the Hyperspectral Imaging System

**Figure 3:**
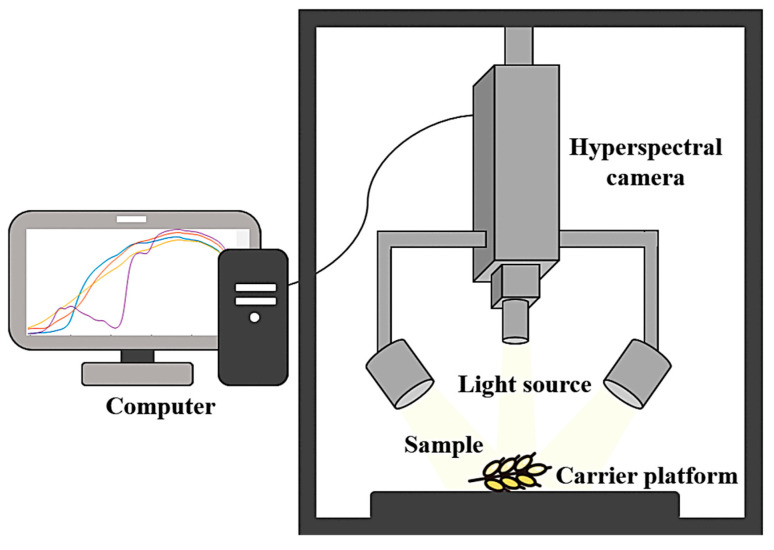
Hyperspectral imaging system.

Figure 3 illustrates the key components of a typical hyperspectral imaging system. The light source illuminates the system across a broad wavelength range, from ultraviolet (200~400 nm) and visible light (400~760 nm) to near-infrared (760~2500 nm) and beyond 2500 nm [27]. Common light sources include xenon lamps (100~1000 W), halogen lamps (10~300 W), light-emitting diodes (ranging from a few watts to tens of watts), and lasers (few milliwatts to tens of watts) [28]. The hyperspectral camera captures both spectral and image data, which are transmitted to a computer for processing and analysis. The ImSpector V10E camera (Spectra Imaging Ltd., Oulu, Finland) commonly used in most laboratories features a CMOS chip with 1280 (spatial) × 1024 (spectral) pixel resolution, a frame rate of 14 fps, a spectral range of 408~1117 nm, and a spectral resolution of 2.8 nm.

### 2.2. Principles of Hyperspectral Imaging Technology

Hyperspectral imaging integrates machine vision and spectroscopy to simultaneously capture spatial (two-dimensional; 2D) and spectral (one-dimensional; 1D) data, enabling nondestructive material characterization with high resolution (5~10 nm spectral bandwidth) across ultraviolet to near-infrared (200~2500 nm) wavelengths [3]. Compared to conventional optical methods, hyperspectral imaging provides superior analytical accuracy by resolving intrinsic material properties through narrow spectral bands [29]. The visible range (400~760 nm) detects color variations and surface features in grains, such as fungal contamination in wheat, identified by chlorophyll degradation at 680 nm [30]. In the near-infrared range (760~2500 nm), hyperspectral imaging utilizes absorption features from molecular vibrations (C-H, O-H, N-H) for quantitative analysis of grain physicochemical properties, including moisture and chemical composition [31].

**Figure 4:**
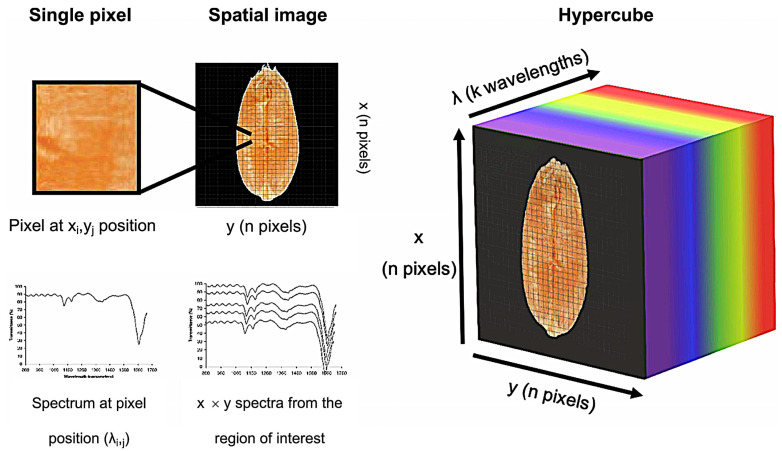
Three-dimension data cube of hyperspectral imaging. Relationship between the spatial resolution (x, y) and the spectral resolution (λ) [34].

Hyperspectral images are three-dimensional data cubes comprising two spatial dimensions (x, y) and one spectral dimension (λ), where each pixel encodes both spatial coordinates and a continuous spectrum (400~2500 nm) [32,33]. As illustrated in Figure 4 [34], the spatial dimensions define pixel positions, while the spectral dimension encodes spectral data [35]. At a given wavelength, pixel grayscale values correlate with analyte-specific absorption, enabling simultaneous quantification of external features (e.g., size, color, shape and surface defects) and internal attributes (e.g., internal defects and chemical composition) [36,37,38]. This dual spatial–spectral capability drives hyperspectral imaging applications in agriculture.

### 2.3. Hyperspectral Imaging Acquisition Mode

Hyperspectral acquisition modes include reflection, transmission and scattering depending on the location of the light source, hyperspectral camera and sample [3]. In reflection mode, the light source and sample are on the same side, and the camera captures light reflected from the sample surface, typically used to assess external grain characteristics such as size, color, and surface defects [39]. In transmission mode, the light source and camera are positioned on opposite sides of the sample, with the camera collecting transmitted light. This mode is used to evaluate internal composition and defects, requiring the sample to have sufficient transmittance [32]. Scattering mode, an extension of transmission mode, is less commonly used.

### 2.4. Hyperspectral Image Acquisition Approaches

**Figure 5:**
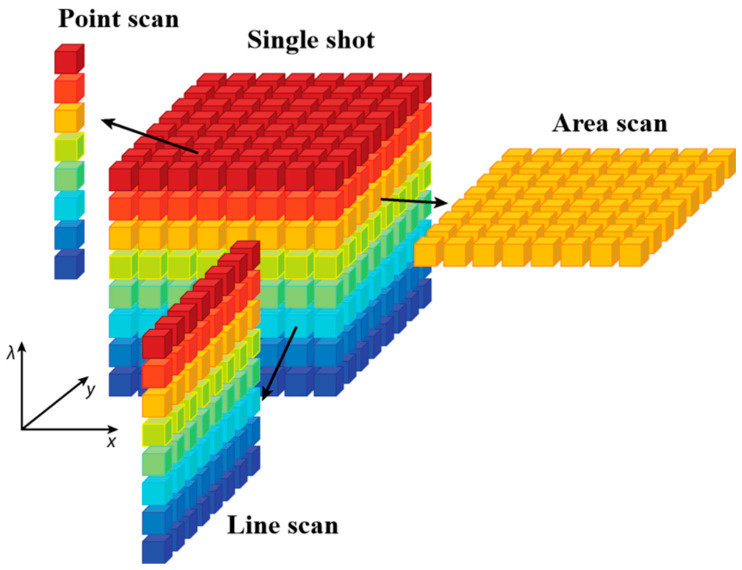
Hyperspectral image acquisition approaches: point scanning, line scanning and area scanning [32].

Figure 5 [32] illustrates the classification of hyperspectral imaging based on acquisition and formation methods into point scanning, line scanning, and area scanning [40]. The x- and y-axes represent the spatial dimensions (width and height of the sample), while the λ-axis represents the spectral dimension (wavelength range). In point scanning, the hyperspectral camera or sample moves along the x- and y-axes, capturing the spectrum of a single pixel at a time, which is then combined to form the hyperspectral image. This approach is well-suited for static, high-precision inspections [32]. Line scanning involves moving the camera or sample along a predefined path to capture the spectrum of all pixels in a line, making it effective for dynamic detection of objects on a conveyor belt [41]. In area scanning, the entire image is captured at each wavelength sequentially, with repeated acquisitions across different wavelengths. The hyperspectral image is constructed by stacking these images, making this method effective for applications requiring data from multiple wavelengths [1,42].

### 2.5. Basic Hyperspectral Imaging Processing Steps

**Figure 6:**
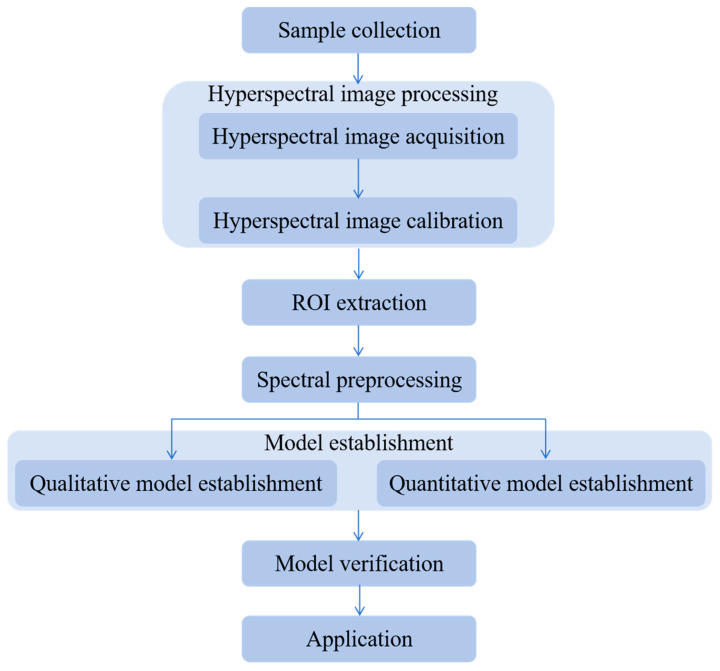
Hyperspectral imaging analysis flow chart.

Following hyperspectral image acquisition (Figure 6), data processing employs ENVI (version 6.0) for image preprocessing and region-of-interest (ROI) extraction, with MATLAB (version R2022a) or Python (version 3.8) for spectral preprocessing, feature extraction, and model establishment. The process consists of three core stages: noise reduction and spectral correction (preprocessing), feature wavelength extraction, and predictive model development [1,43].

Preprocessing mitigates artifacts from environmental interference (e.g., ambient light fluctuations) and instrument noise, enhancing signal-to-noise in raw spectra [44,45]. Feature selection then isolates relevant characteristic wavelengths, reducing data dimensionality while preserving predictive accuracy [1]. Machine learning models then associates spectral features with target attributes. Qualitative analysis focuses on the identification and classification of substances. In contrast, quantitative analysis relates specific sample indicators to spectral data, aiming to predict substance characteristics. This workflow enables real-time, nondestructive grain quality assessment. Commonly used preprocessing, feature selection and modeling methods are listed in Table 1.

As a representative example, wheat samples with varying quality states (e.g., healthy, contaminated) are collected to ensure sample diversity. A hyperspectral imaging system captures image data of each sample, with each pixel containing both visual (e.g., color, texture) and chemical information (e.g., moisture content, protein levels). Image calibration eliminates external environmental factors. Regions of interest (ROIs), such as wheat kernels, are extracted to exclude irrelevant data. During the spectral preprocessing, denoising techniques are applied to improve data accuracy. A qualitative model is developed to distinguish healthy from contaminated wheat, while a quantitative model quantifies indicators like moisture and protein content. Once validated, these models are applied in real time, enabling rapid classification of wheat as “acceptable” or “unacceptable” and the calculation of specific values (e.g., moisture, protein content), facilitating fast, real-time grain quality and safety assessments.

## 3. Application of Hyperspectral Imaging in Grain Detection

Over the past five years, hyperspectral imaging has seen widespread adoption in the grain industry [34,48]. Its applications span disease detection, quality assessment, physicochemical property analysis, pesticide residue detection, and geographical origin assessment. The following sections explore each application in detail.

### 3.1. Disease Detection

Grain diseases can severely affect crop quality and yield [49]. Traditional methods, such as visual inspection or laboratory testing, are time-consuming and often imprecise [50,51]. Hyperspectral imaging offers a rapid, nondestructive alternative, detecting diseases by identifying spectral features associated with pathological changes in grain structure [52,53,54]. As shown in Table 2, recent studies highlight hyperspectral imaging as an effective tool for detecting various grain diseases. Specific spectral signatures in the visible and near-infrared wavelengths have been identified as reliable indicators of disease presence, enabling early-stage detection and intervention.

#### 3.1.1. Hyperspectral Imaging in Wheat Disease Detection and Algorithmic Challenges

Hyperspectral imaging in grain disease detection highlights the complex interaction between spectral biochemistry, advanced algorithms, and the unique physiological responses of grains. Recent advances highlight how hyperspectral imaging reveals pathological changes, enabling early detection of fungal infections and mycotoxins. Studies have demonstrated that near-infrared hyperspectral imaging (900~1700 nm), coupled with Partial Least Squares Discriminant Analysis (PLS-DA), achieves over 92% accuracy in quantifying deoxynivalenol (DON) contamination in wheat infected with Fusarium head blight (FHB). This high accuracy is attributed to spectral features associated with DON-induced protein denaturation at 1660 nm and chlorophyll degradation at 680 nm [30]. However, linear discriminant models struggle with multi-pathogen infections, such as when oats are co-infected with Fusarium, Aspergillus, and Penicillium, leading to significant classification errors. To address this constraint, Liang et al. [57] fused the Faster Region-based Convolutional Neural Network (Faster R-CNN) with hyperspectral feature bands to achieve 99% accuracy using nonlinear decision boundaries. These findings highlight the evolution of hyperspectral techniques, with algorithmic innovations addressing the complexities of multifactorial grain diseases.

#### 3.1.2. Hyperspectral Imaging in Maize Disease Detection and Specific Challenges

The starch-rich matrices in maize introduce spectral overlap at 980 nm (amylose) and 1450 nm (amylopectin), confounding traditional Principal Component Analysis (PCA) -based workflows. To address this problem, a study integrated Vis-NIR hyperspectral imaging with Competitive Adaptive Reweighted Sampling (CARS) to isolate aflatoxin B1-specific bands to achieve 98.2% classification accuracy using K-Nearest Neighbor (KNN) [59]. In addition, the orientation of maize kernels can alter spectral reflectance, necessitating One-dimensional Convolutional Neural Network (1D-CNN) architectures to mitigate spatial variability [60]. This approach is analogous to advancements in leaf-based crop detection, where deep CNN models have been employed to identify plant diseases from leaf images [66]. This underscores the broader applicability of CNNs in detecting subtle pathological features. Unlike wheat research, this challenge highlights the need for crop-specific algorithmic refinements to address the complex spectral signatures arising from biochemical composition and physical morphology in grain disease detection.

#### 3.1.3. Emerging Applications in Other Grains: Oats, Barley, and Millet

While wheat and maize dominate hyperspectral imaging research, emerging applications in oats, barley, and millet underscore the technology’s adaptability. For instance, Teixido-Orries et al. [61] used NIR hyperspectral imaging with first derivative preprocessing to detect T-2 and HT-2 toxins in oats, identifying key wavelengths at 1038, 1110, and 1393 nm. Their KNN model achieved 94.1% accuracy, suggesting hyperspectral imaging’s effectiveness in detecting these toxins under laboratory conditions. In millet, Nie et al. [63] pioneered the detection of ergosterol and DON using wavelet-transformed spectral data and attention-LSTM networks. Millet’s compact kernel structure amplifies spectral noise, thus requiring wavelet transform for denoising. Similarly, Su et al. [62] automated DON quantification in barley using iterative feature selection (ISSPA), compensating for barley’s lower near-infrared penetration compared to wheat. These studies illustrate the expanding scope and adaptability of hyperspectral imaging for detecting toxins in a variety of grains.

#### 3.1.4. Hyperspectral Imaging for Contaminant Detection and Disease Severity Quantification

Hyperspectral imaging extends beyond biological pathogen detection to include industrial contaminants, such as paraffin in rice. Wang et al. [64] developed an efficient method based on hyperspectral imaging for rapid detection of paraffin contamination, demonstrating its utility in ensuring grain purity and safety. Beyond detection, hyperspectral imaging has been instrumental in quantifying disease severity and screening for resistance traits. Xue et al. [67] used proximal hyperspectral imaging to quantify the severity of rice spikelet rot, monitoring disease progression at the organ level. Through spectral feature extraction, they distinguished diseased from healthy rice spikelet, highlighting hyperspectral imaging’s potential in precision agriculture. This study reinforces the utility of hyperspectral imaging as a rapid, non-invasive technique for distinguishing healthy from contaminated crops, eliminating the need for extensive laboratory procedures.

#### 3.1.5. Challenges and Limitations

Hyperspectral imaging has emerged as a pivotal tool in food safety and quality assessment, particularly for detecting fungal contamination, evaluating disease severity, and quantifying mycotoxin in cereal grains. Recent advancements have shown its potential in nondestructive testing, enabling rapid screening and real-time decision-making in food production and processing. The integration of hyperspectral imaging with advanced machine learning models, especially deep learning algorithms, has enhanced its potential for high-precision prediction and classification tasks. However, challenges remain in standardizing hyperspectral imaging protocols, particularly regarding spectral calibration, data preprocessing, and model training. Future research should focus on improving the robustness, scalability, and consistency of hyperspectral imaging systems, while also exploring new applications in food safety, traceability, and authenticity verification.

### 3.2. Quality Assessment

In recent years, hyperspectral imaging has gained significant traction in agriculture, particularly for grain quality assessment [68]. It enables the detection of key attributes such as morphology, composition, damage, and contamination by capturing detailed spectral information [69,70]. This allows for accurate grain classification and the identification of internal defects and impurities, supporting real-time quality monitoring [71]. A summary of seminal research in this area is presented in Table 3, covering variety identification, physical damage detection, adulteration analysis, and assessment of freshness and germination.

#### 3.2.1. Variety Identification

The ability of hyperspectral imaging to differentiate grain varieties marks a major advancement for the agricultural and food industries [79,82]. Recent studies demonstrate high classification accuracy, particularly in wheat, through standardized workflows that combine spectral preprocessing (e.g., SG, MSC), feature selection (e.g., CARS, SPA), and machine learning models (e.g., CNN, PLS-DA). These methods effectively resolve subtle spectral variations linked to varietal genetics, physicochemical properties, and structural integrity [73,78]. Beyond wheat, hyperspectral imaging has shown promise in identifying other grains. For instance, the use of a novel one-dimensional convolutional neural network (CAM-TM-1DCNN) embedded with a channel attention module (CAM) and transformer module (TM) to replace traditional convolutional neural networks (CNN), enables accurate identification of flaxseed varieties [77]. In the prediction of millet varieties, the application of the Gradient tree boosting algorithm achieved a classification accuracy of 99% [76]. These advances underscore the potential of deep learning and hybrid models in achieving high-precision classification of diverse agricultural products.

#### 3.2.2. Mechanical Damage and Adulteration Detection

Detection of mechanical damage and adulteration exemplifies the shift from traditional linear models to hybrid and deep learning approaches [40,88]. For example, in wheat kernel analysis, advanced machine learning models like least squares-support vector machines (LS-SVM) applied to near-infrared (NIR) spectra have achieved 100% accuracy in identifying mechanical damage [39]. In contrast, corn seed damage demanded ResNeSt_E architectures to mitigate spatial heterogeneity, achieving 99% accuracy by fusing Vis-NIR data [87].

The detection of adulterated grains has become a crucial application of hyperspectral imaging in the food industry, significantly impacting grain quality and market value [89,90]. Studies on sorghum [84] and coarse grain flours [83] highlighted the superiority of PLS-DA and CARS or PCA for isolating adulterant-specific wavelengths. In particular, the detection of sorghum adulteration is critical because the purity of sorghum directly affects the quality and flavor of baijiu.

#### 3.2.3. Intrinsic Biochemical Changes Monitoring

Unlike conventional surface analysis techniques, hyperspectral imaging allows for the characterization of intrinsic biochemical changes in cereal grains, offering a critical framework for nondestructive detection of germinated wheat kernels. Mildly sprouted wheat kernels, which are visually similar to intact kernels, can compromise product quality. Zhang et al. [86] used hyperspectral data from both sides of the wheat kernel to assess its condition comprehensively, achieving promising results for quality control applications in agriculture. Similarly, a Random Forest (RF) model correctly identified germinating sorghum with 91% accuracy [85]. These studies underscore the significance of hyperspectral imaging in monitoring essential parameters such as germination, freshness, and varietal characteristics, ensuring grain quality.

#### 3.2.4. Challenges and Limitations

While hyperspectral imaging, combined with advanced machine learning algorithms, has significantly improved grain quality assessment, several challenges remain. These include issues with data standardization, spectral calibration, and the need for robust models capable of handling large, diverse datasets. Additionally, the integration of hyperspectral imaging into routine grain quality control practices faces hurdles in terms of scalability, cost, and the complexity of real-time data processing. Despite these challenges, the potential for hyperspectral imaging to transform food safety protocols and enhance supply chain transparency is substantial. Looking ahead, integration with Internet of Things (IoT) systems and real-time analysis platforms could enable continuous quality assessments during grain processing, ensuring the use of only high-quality grains in food production.

### 3.3. Physicochemical Property Detection

Hyperspectral imaging has revolutionized the nondestructive assessment of key physicochemical properties in grains, such as moisture content, protein, and fat content [91,92]. Regression models, developed using modeling algorithms, can predict the concentrations of these attributes, which are crucial for grain selection and grading. As nutritional value is closely linked to these internal properties, their assessment is essential for various applications [41,93]. Table 4 summarizes hyperspectral imaging applications over the past five years. It highlights the integration of machine learning and spectral preprocessing to address grain-specific challenges, though issues with model generalization and scalability persist.

#### 3.3.1. Oil Content Detection

Oil content is a critical indicator of grain quality. Traditional detection methods are time-consuming and rely on complex chemical reagents. In contrast, hyperspectral imaging technology allows for rapid and accurate prediction of oil content by analyzing the grains’ reflection spectra. This method has been successfully applied to corn and sorghum. Zhang et al. [94] integrated hyperspectral imaging with an attention based convolutional neural network regression (ACNNR) to predict oil content of individual corn kernels, outperforming traditional PLSR models [95]. The efficacy of ACNNR stems from its direct utilization of unprocessed spectral data, eliminating the need for manual preprocessing, feature extraction or selection. This approach is robust against measurement inaccuracies caused by particle heterogeneity, a challenge less significant in sorghum. However, PLSR remains favored for sorghum due to its simplicity and lower computational demands. This highlights the trade-off between accuracy and practicality.

#### 3.3.2. Moisture Content Detection

Moisture content significantly affects the storage and processing of grains [97]. Hyperspectral imaging, coupled with chemometrics, has emerged as a widely adopted technique for moisture detection [109]. In recent years, its application has evolved from static measurement [98,100,110] to real-time monitoring during processing. For example, Zhang et al. [97] used hyperspectral imaging to determine and visualize the moisture content and pasting degree of rice during soaking and cooking. It supported the development of intelligent rice processing systems. These studies demonstrate the technology’s practical potential. Additionally, moisture content correlates with other quality indicators, such as textural attributes like hardness, offering a more comprehensive understanding of grain quality and its intrinsic properties.

#### 3.3.3. Comprehensive Monitoring of Grain Quality Indicators

Hyperspectral imaging technology can monitor a range of grain quality indicators, including critical constituents like starch [106], protein [20,99], fat [100,101], total acid [102], tannin content [103], reducing sugar [102], nutritious substance [104,105] and alcohol content [107]. These components directly influence the nutritional value and functional performance of grains during food processing [111]. By capturing spectral signatures associated with these constituents, the technology enables comprehensive analysis of their interrelationships. This capability surpasses the limitations of single-parameter tests, improving the accuracy of grain quality assessments and supporting the analysis of multiple grain attributes [108,112].

#### 3.3.4. Challenges and Limitations

Despite significant progress in hyperspectral imaging for grain analysis, several challenges hinder its widespread adoption. First, quality metrics for different grain types at various stages of distribution are often inconsistent. This requires careful selection of relevant samples and metrics tailored to each analysis context. Additionally, determining appropriate preprocessing methods, sample segmentation techniques, and modeling algorithms remains crucial. This process often involves iterative testing to identify the most effective model configurations. Second, the high costs and complex operation of hyperspectral imaging systems limit their deployment in production environments. The large computational demands for data processing further necessitate the optimization of spectral data processing algorithms to improve efficiency and accuracy. Overcoming these challenges is essential for fully realizing the potential of hyperspectral imaging in grain inspection.

### 3.4. Pesticide Residue Detection

The use of pesticides can effectively mitigate the impact of insects and diseases on crops, promoting healthy grain growth [109]. However, improper application presents significant risks to human health [113]. Therefore, developing rapid and accurate analytical methods for detecting pesticide residues persists as a pressing challenge in ensuring safety across global agricultural and food production systems. In contrast to traditional techniques like gas chromatography (GC) and high-performance liquid chromatography (HPLC), hyperspectral imaging offers notable advantages. These include speed and nondestructive analysis, making it well-suited for modern agricultural needs. Although hyperspectral pesticide detection has been extensively studied in fruits and vegetables, its application to grains is still in its early stages.

Most current pesticide residue detection methods using hyperspectral technology employ feature selection algorithms to select specific wavelengths for agricultural products. Traditional machine learning models are then trained to predict pesticide residue concentrations based on these wavelengths, enabling visualization of residue distributions [113]. However, most studies focus on a single pesticide and overlook the prevalence of multiple pesticides in agricultural practices. An exception is Bian et al. [114], who used hyperspectral imaging combined with machine learning to detect residues from three different insecticides in cantaloupes. This study not only validates the feasibility of multi-residue detection but also provides a methodological framework adaptable to other agricultural products.

**Figure 7:**
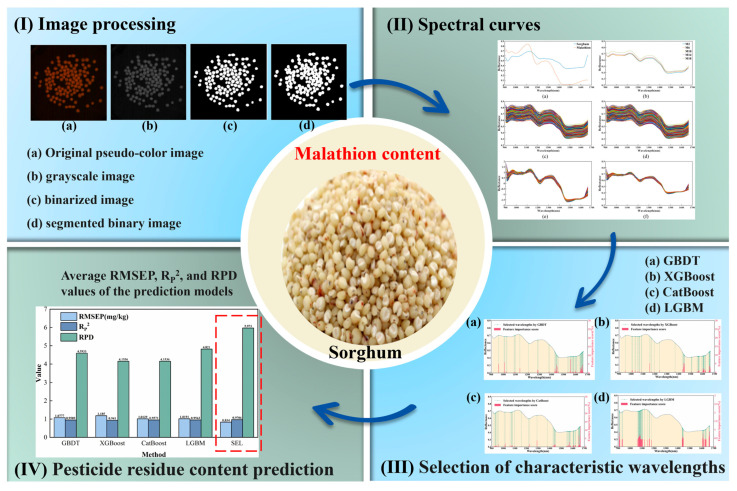
(**I**). Hyperspectral image processing. (**a**) Original pseudo-color image; (**b**) grayscale image; (**c**) binarized image; (**d**) segmented binary image. (**II**). Spectral curves: (**a**) average raw spectral of pure malathion and sorghum; (**b**) average raw spectral of M2, M6, M10, M14, M18; (**c**) raw spectral; (**d**) spectral after SG preprocessing; (**e**) spectral after SNV preprocessing; (**f**) spectral after MSC preprocessing. (**III**). Distribution of characteristic wavelengths and contribution rates identified by four feature selection methods: (**a**) GBDT, (**b**) XGBoost, (**c**) CatBoost, and (**d**) LGBM. (**IV**). Average RMSEP, R_P_^2^, and RPD values of the prediction models [116].

The success of hyperspectral imaging for pesticide detection depends heavily on advanced data processing techniques [115]. For instance, Peng et al. [116] successfully detected malathion residues in sorghum by integrating hyperspectral imaging with stacked machine learning (SEL) model for the first time. This approach bridges a significant gap in residue detection for cereals and provides a foundational framework for future research. Figure 7 illustrates the detection process. Experimental results revealed that, for both full spectrum and characteristic wavelengths, the SEL model outperformed other models in prediction accuracy. Notably, the CatBoost-SEL model demonstrated the highest predictive performance. Hu et al. [117] further employed this model for the classification of pesticide residues in sorghum, highlighting its superior capabilities in this context.

This study highlights that hyperspectral imaging for detecting pesticide residues in grains is still in its early stages, especially compared to established methods for fruits and vegetables. A major challenge is the impact of grain surface characteristics (e.g., roughness and uneven coloration) on spectral data quality. This limits model generalizability. Additionally, outer coatings like the husk and bran further complicate the detection of surface pesticide residues [2]. The broad spectrum of pesticide residues and overlapping spectral features also hinder accurate detection. While stacked machine learning models improve prediction accuracy, their high computational demands limit real-time application. Future research should focus on: (1) optimizing spectral preprocessing to reduce surface interference; (2) developing multi-task learning models to detect multiple pesticide residues simultaneously; (3) exploring lightweight machine learning algorithms for real-time detection; and (4) integrating other nondestructive testing techniques to enhance detection accuracy and reliability.

### 3.5. Geographical Origin Assessment

The geographical origin of grain is crucial for ensuring quality, safety, and consumer protection. Given growing food safety concerns globally, assessing geographical origin has become vital in the supply chain [118]. In this context, hyperspectral imaging has emerged as a promising tool for evaluating food products, particularly for determining grain origin. Its advantages, including simplicity, speed, efficiency, and environmental sustainability, make it an increasingly viable solution.

#### 3.5.1. Deep Learning Decodes Geographical Signatures in Grain Hyperspectral Fingerprints

**Figure 8:**
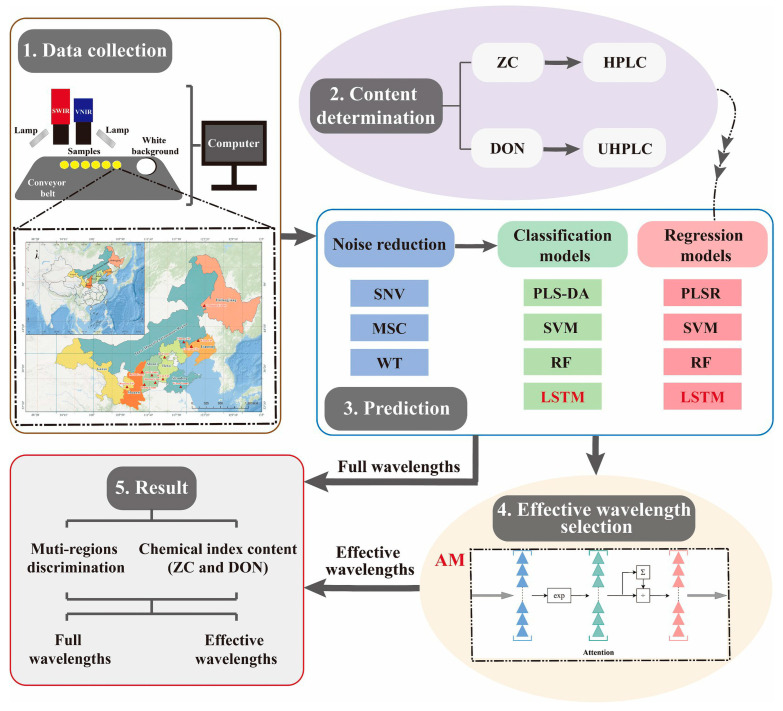
Workflow of the proposed approach, comprising spectral data acquisition, chemical composition analysis, model prediction (encompassing data denoising, origin discrimination, and content regression), and feature wavelength selection using the attention mechanism (AM) algorithm [63].

Hyperspectral imaging captures spectral fingerprints that reflect the biochemical and physical properties of grains, which vary by geographical origin. For example, Nie et al. [63] collected 600 millet samples from 12 production regions across China and developed a deep learning model, achieving over 99% accuracy. This highlights the potential of hyperspectral imaging for non-destructive grain origin determination, particularly when paired with deep learning to enhance prediction accuracy. The detection process is illustrated in Figure 8.

#### 3.5.2. Exploration of Hyperspectral Imaging for Origin Traceability in Special Grains

Beyond common grains, hyperspectral imaging shows promise for special grains. Their spectral properties may differ significantly due to variations in physical structure and biochemical composition. Applying this technology to such grains provides insights into its potential for adapting to a wider range of crops. Choi et al. [119] applied hyperspectral imaging to discriminate the geographical origin of chia seeds, achieving a classification accuracy of 91.11%. These studies demonstrate hyperspectral imaging’s ability to detect subtle compositional differences that can be crucial for traceability.

#### 3.5.3. Machine Learning Integration for Enhanced Geographic Origin Classification of Grains

The integration of hyperspectral imaging with machine learning has greatly improved grain classification based on geographical origin. Edris et al. [120] employed unsupervised clustering algorithms to analyze hyperspectral images of rice, successfully identifying its authenticity and geographical origin. Combined with robust clustering techniques, hyperspectral imaging can efficiently process large datasets and detect patterns without labeled training data. This approach offers a cost-effective solution for food traceability. Furthermore, Van De Steene et al. [121] developed fingerprinting methods for assessing rice origin and variety, enhancing HSI accuracy through data fusion. These advancements demonstrate the synergy between hyperspectral imaging and machine learning in addressing complex classification tasks. The preprocessing algorithms used in these studies are summarized in Table 5.

#### 3.5.4. Challenges and Limitations

The ability to accurately determine the geographical origin of grains has significant implications for food traceability, authenticity, and consumer trust. Hyperspectral imaging provides a rapid, nondestructive, and precise method for verifying grain origin. It also supports regulatory compliance and quality control in the food supply chain. Despite its potential, several challenges remain. A key limitation is the need for extensive calibration across diverse regions and variable environmental conditions. Grains’ spectral signatures are affected by factors such as seasonal variations, cultivation practices, and post-harvest processing, leading to significant variability. Therefore, large-scale hyperspectral data repositories are necessary to develop robust origin prediction models. Additionally, while hyperspectral imaging shows promise in laboratory settings, its application in real-world environments, such as grain storage, markets, and distribution centers, requires further optimization for large-scale use.

## 4. Conclusions and Perspectives

Hyperspectral imaging combines imaging and spectroscopy to capture both spatial and spectral data, enhancing the quality and safety assessment of agricultural products. It enables evaluation of attributes such as species identification, nutritional content, contamination detection, freshness, and geographic origin. These features make it a rapid, non-destructive, and cost-effective tool for agricultural analysis. However, hyperspectral imaging faces challenges, including the impact of environmental factors, high system costs, complex operation, and large data volumes. Additionally, the lack of standardized protocols hampers comparison and validation across studies, limiting broader adoption.

Future research should focus on optimizing data processing methods and algorithms for more efficient and rapid analysis. Tailored algorithmic improvements for various imaging environments will be crucial for enhancing data analysis precision. The large and costly nature of current laboratory-based hyperspectral image systems also calls for the development of more portable, cost-effective alternatives. Recent advancements in portable systems integrated with environmental sensors show promise for broader food industry applications. Furthermore, multispectral imaging and RGB-based hyperspectral image reconstruction offer potential solutions to the high cost and redundancy of hyperspectral data. Challenges such as insufficient sample diversity, limited data sets, and a lack of standardized protocols in food hyperspectral research highlight the need for dedicated institutions to develop a multi-category food hyperspectral database and promote resource sharing. Standardized calibration procedures for hyperspectral imaging systems will enhance the consistency and reliability of spectral data across studies. Additionally, uniform protocols for spectral data preprocessing and acquisition will improve reproducibility and comparability. Adopting standardized reporting guidelines, detailing methods, instrumentation, calibration, and data processing, will further enhance transparency and result reproducibility. In the long term, deep learning algorithms and hyperspectral databases will enhance prediction accuracy. Further exploration of hyperspectral imaging for pesticide residue detection and its integration with complementary technologies may provide innovative, nondestructive solutions for agricultural analysis.

## Figures and Tables

**Table 1:** Statistical tables of common spectral preprocessing, feature selection and modeling methods.

	Method	Function	Reference
Preprocessing	Savitzky–Golay Filtering (SG)	Smoothing and noise reduction via polynomial fitting.	[3,46]
	Derivative Method	Enhance spectral features by calculating 1st/2nd derivatives.	
	Multiplicative Scatter Correction (MSC)	Correct scattering effects caused by uneven surfaces.	
	Mean Centering (MC)	Subtract mean spectrum to emphasize variations.	
	Orthogonal Signal Correction (OSC)	Remove orthogonal noise unrelated to target variables.	
	Standard Normal Variate (SNV)	Normalize spectra by row-wise scaling.	
Feature Selection	Principal Component Analysis (PCA)	Dimensionality reduction by projecting data onto orthogonal axes.	[28,32,47]
	Competitive Adaptive Reweighted Sampling (CARS)	Select optimal wavelengths via adaptive weighting.	
	Successive Projections Algorithm (SPA)	Minimize wavelength redundancy via vector projection.	
	Random Frog (RF)	Stochastic wavelength selection based on probability.	
	Genetic Algorithm (GA)	Evolutionary optimization of wavelength subsets.	
	Uninformative Variables Elimination (UVE)	Remove non-informative wavelengths via stability analysis.	
	Support Vector Machine (SVM)	Classify samples via hyperplane optimization.	
	K-Nearest Neighbor (KNN)	Assign labels based on proximity in feature space.	
	Artificial Neural Network (ANN)	Non-linear classification via layered neuron networks.	
	Linear Discriminant Analysis (LDA)	Maximize inter-class variance for separation.	
	Partial Least Squares-Discriminant Analysis (PLS-DA)	Supervised classification combining PLS and LDA.	
	Support Vector Regression (SVR)	Predict continuous variables via kernel-based regression.	
	Stepwise Linear Regression (SLR)	Iteratively select variables for linear modeling.	
	Partial Least Squares Regression (PLSR)	Relate spectral data to reference values via latent variables.	
	Multiple Linear Regression (MLR)	Multi-variable linear regression for rapid prediction.	

**Table 2:** Application of hyperspectral imaging in grain disease detection.

Grain	Contaminant	Spectral Range	Preprocessing Method	Model Types	Model Accuracy	Reference
Maize	Maize ear rot pathogens	NIR	PCA	PLS-DA	93.75%	[55]
Wheat	FHB	NIR	SNV	PLS-DA	>92%	[30]
Wheat	FHB	Vis-NIR	ReliefF, SFLA, UVE, RF	ASSDN	98.31%	[56]
Wheat	FHB	NIR	MSC, PCA	Faster R-CNN	99.00%	[57]
Wheat	DON	NIR	SG, NG, SNV, MSC	ANN	85.80%	[58]
Maize	Aflatoxin B1	Vis-NIR	MSC, SNV, SG	KNN	98.20%	[59]
Maize	Fungal contamination	Vis-NIR	SNV, SG	1D-CNN	>98%	[60]
Oat	T-2 and HT-2 toxins	NIR	SNV, First derivative	KNN	94.10%	[61]
Barley	DON	NIR	CARS, ISSPA	LWPLSR	R_P_^2^ = 0. 728 RMSEP = 3.802	[62]
Millet	Ergosterol and deoxynivalenol	Vis-NIR	WT	WT-LSTM	R^2^ > 0.95 RPD > 3.50	[63]
Rice	Industrial paraffin contamination	NIR	MSC, SNV, SG	PLSR	R^2^ = 0. 9338 RMSE = 0.090%	[64]
Wheat	DON	NIR	SNV	LDA	100%	[65]

ASSDN, Architecture self-search deep network; ANN, Artificial Neural Network; CARS, Competitive Adaptive Reweighted Sampling; CNN, Convolutional Neural Network; DON, deoxynivalenol; FHB, Fusarium head blight; Faster R-CNN, Faster Region-based Convolutional Neural Network; ISSPA, Iterative Selection of Successive Projections Algorithm; KNN, K-Nearest Neighbors; LDA, Linear Discriminant Analysis; LWPLSR, Locally Weighted Partial Least Squares Regression; MSC, Multiplicative Scatter Correction; NIR, Near Infrared; PCA, Principal Component Analysis; PLS-DA, Partial Least Squares Discriminant Analysis; PLSR, Partial Least Squares Regression; RF, Random Frog; R_P_^2^, Correlation coefficient of prediction; RMSEP, Root Mean Squared Error of Prediction; R^2^, Coefficient of determination; RMSE, Root Mean Squared Error; RPD, Ratio of Performance to Deviation; SG, Savitzky–Golay Filtering; SNV, Standard Normal Variate; Vis-NIR: Visible and Near-Infrared; WT, Wavelet Transformation; 1D-CNN, One-dimensional Convolutional Neural Network.

**Table 3:** Application of hyperspectral imaging in grain quality assessment.

Grain	Quality Parameter	Spectral Range	Preprocessing Method	Model Types	Model Accuracy	Reference
Maize seed	Variety identification	NIR	SG, SNV, SSAE	SSAE-CS-SVM	95.81%	[72]
Cereal	Variety identification	Vis-NIR	PCA	BPNN	90%	[73]
Pulse flours	Variety identification	Vis-NIR	PCA	PLS-DA	100%	[74]
Grain	Variety identification	397–1004.5 nm	DBSCAN, MD, PCA, IVSO, CARS	BPNN	99.00%	[75]
Millet	Variety identification	NIR	SG, SVN, PCA	Gradient tree boosting	99.4%	[76]
Flaxseed	Variety identification	NIR	SG, PCA, LDA, SPA, CARS	CNN	95.26%	[77]
Sorghum	Variety identification	NIR	IF, MSC, CR, OSC	AlexNet	95.91%	[78]
Wheat	Variety identification	Vis-NIR	SG, CARS	DLFM	92.87%	[79]
Wheat	Variety identification	Vis-NIR, SWIR	SVN, SG, LDA, SVM, ANN	LDA-SVN	94.93%	[80]
Wheat	Variety identification	Vis-NIR	Not mentioned	3D CNN	98.40%	[81]
Wheat	Variety identification	Vis-NIR	SG, MSC, CARS	BP	92.29%	[82]
Coarse grain flours	Adulteration level	NIR	PCA, SPA, CARS	PLS-DA	>94.8%	[83]
Sorghum	Adulteration level	NIR	PCA	PLS-DA	91.00%	[84]
Sorghum	Adulteration level	NIR	IF, MSC, CARS, SPA	DF	>91%	[85]
Wheat	Sprouted kernels	Vis-NIR	SG, CARS	DF	89%	[86]
Wheat	Mechanical damage	NIR	PCA, SPA	LS-SVM	100%	[39]
Corn seeds	Mechanical damage	Vis-NIR	PCA, KPCA, FA	ResNeSt_E	99.00%	[87]

3D CNN, 3D Convolutional Neural Network; ANN, Artificial Neural Networks; BPNN, Backpropagation Neural Network; BP, back-propagation; CARS, Competitive Adaptive Reweighted Sampling; CNN, Convolutional Neural Network; CR, Continuum Removal; DBSCAN, Density-Based Spatial Clustering of Application with Noise; DF, deep forest; DLFM, Deep Learning Feature Fusion Model; FA, factor analysis; IF, Isolated Forest; IVSO, Iterative Variable Subset Optimization; KPCA, Kernel PCA; LDA, Linear Discriminant Analysis; LDA-SVN, Linear Discriminant Analysis-Standard Normal Variate; LS-SVM, Least Square-Support Vector Machine; MD, Mahalanobis distance; MSC, Multiplicative Scatter Correction; OSC, Orthogonal Signal Correction; PCA, Principal Component Analysis; PLS-DA, Partial Least Squares Discriminant Analysis; SG, Savitzky–Golay Filtering; SNV, Standard Normal Variate; SPA, Successive Projections Algorithm; SSAE-CS-SVM, stacked sparse autoencoder combined with a cuckoo search optimized support vector machine; SVN, Standard Normal Variate.

**Table 4:** Application of hyperspectral imaging in physicochemical property detection.

Grain	Property	Spectral Range	Preprocessing Method	Model Types	Model Accuracy	Reference
Maize	Oil content	NIR	SNV, SG, SG1, SG2, SPA, CARS, PCA, CNN	ACNNR	R_p_^2^ = 0.9198	[94]
Sorghum	Oil content	NIR	SNV	PLS	SEC = 0.25% SEP = 0.26%	[95]
Maize seed	Moisture content	NIR	SG	PLSR-UVE	R_p_ = 0.970 RMSEP = 0.454%	[96]
Rice	Moisture content	NIR	SG, Second derivative	PLSR	R^2^ = 0.9673 RMSE = 0.5300	[97]
Rice	Moisture content	NIR	CARS, SPA	PLSR	R_p_^2^ = 0.9650 RMSEP = 0.0031	[31]
	Fatty acid content				RP2 = 0.8573 RMSEP = 1.6956	
Fermented grains	Moisture content	NIR	SNV, MSC, CARS, SPA	XGBoost	R_p_^2^ = 0.9757 RMSEP = 0.0442 g/100 g	[98]
	Acidity				R_p_^2^ = 0.9941 RMSEP = 0.0216 mmol/10 g	
Rice	Protein content	Vis-NIR	Denoising and sharpening	BPNN	R^2^ = 0.9516 RMSE = 0.3492	[20]
Wheat	Protein content	NIR	MSC, CARS	SEL	R_p_^2^ = 0.9939 RMSEP = 0.0116 g/kg	[99]
Sorghum	Protein content	NIR	PCA, SPA	BP-GA	RPD = 5.1716 AB_RMSE = 0.0916 g/100 g	[100]
	Fat content		UVE, SPA	CF	RPD = 12.9724 AB_RMSE = 0.0243 g/100 g	
Wheat	Protein content	NIR	CARS, SPA	AdaBoost-SVR	R_p_^2^ = 0.9789	[101]
	Fat content				R_p_^2^ = 0.9651	
Fermented grains	Total acid	Vis-NIR	MSC, CARS, SPA	CF	R_p_^2^ = 0.9988 RMSEP = 0.0278 mmol/10 g	[102]
	Reducing sugar		SNV, CARS, SPA	PSO-SVR	R_p_^2^ = 0.9987 RMSEP = 0.0287 g/100 g	
Grains	Tannin content	NIR	DT, IVSO-VIP	DF	R_p_^2^ = 0.9922 RMSEP = 0.0222 g/100 g	[103]
Wheat	Micronutrients	Vis-NIR	First derivative, Second derivative, COE, VN, MMN, MSC	PSLR	r^2^ > 0.70%	[104]
Wheat	Nutrient	Vis-NIR	SG	SLR	R^2^ > 0.6	[105]
Sorghum	Amylose contents	NIR	MSC, KS, PCA, SPA	CF	85.33%	[106]
	Amylopectin contents				92.00%	
Fermented grains	Starch content	Vis-NIR	SNV, CARS, SPA, PCA	SVM-AdaBoost	R_p_^2^ = 0.9976 RMSEP = 0.0992	[107]
	Alcohol content			XGBoost	R_p_^2^ = 0.9969 RMSEP = 0.0605	
Multigrain flour mixes	Ingredient quantification	Vis-NIR	PCA	LDA-QDC	>90%	[108]

BPNN, Backpropagation Neural Network; BP-GA, Backpropagation-Genetic Algorithm; CARS, Competitive Adaptive Reweighted Sampling; CF, Cascade Forest; CNN, Convolutional Neural Network; COE, Constant Offset Elimination; DF, deep forest; IVSO, Iterative Variable Subset Optimization; KS, Kennard-Stone; LDA-QDC, Linear Discriminant Analysis-Quadratic Discriminant Analysis; MMN, Min–Max Normalization; MSC, Multiplicative Scatter Correction; PCA, Principal Component Analysis; PLSR, Partial Least Squares Regression; R^2^, Coefficient of determination; R_P_^2^, Correlation coefficient of prediction; RMSE, Root Mean Squared Error; RMSEP, Root Mean Squared Error of Prediction; RPD, Ratio of Performance to Deviation; SEC, Standard Error of Calibration; SEL, Stacked Ensemble Learning; SEP, Standard Error of Prediction; SG, Savitzky–Golay Filtering; SLR, Stepwise Linear Regression; SPA, Successive Projections Algorithm; SVN, Standard Normal Variate; SVR, Support Vector Regression; UVE, Uninformative Variable Elimination; VN, vector normalization.

**Table 5:** Application of hyperspectral imaging in geographical origin assessment.

Grain	Spectral Range	Preprocessing Method	Model Types	Model Accuracy	Reference
Millet	Vis-NIR	WT	WT-ALSTM	99.40%	[63]
Chia seeds	Vis-NIR	MSC	PLS-DA	91.11%	[119]
Rice	Vis-NIR	MSC, PCA	K-means	Silhouette coefficient of three rice samples were 0.5169, 0.5433 and 0.4964.	[120]
Rice	Vis-NIR	PCA	GP-LVM	>90% (except Thailand and Vietnam samples)	[121]

GP-LVM, Gaussian Process Latent Variable Model; MSC, Multiplicative Scatter Correction; PCA, Principal Component Analysis; PLS-DA, Partial Least Squares Discriminant Analysis; Vis-NIR; Visible and Near-Infrared; WT, Wavelet Transformation; WT-ALSTM, Wavelet Transformation-Attention Mechanism Long Short-term Memory.

## Data Availability

Not applicable.

## Abbreviations

1D-CNN: One-dimensional Convolutional Neural Network; AM: Attention Mechanism; ANN: Artificial Neural Networks; ASSDN: Architecture Self-search Deep Network; BP: Backpropagation; BPNN: Backpropagation Neural Network; BP-GA: Backpropagation-Genetic Algorithm; CARS: Competitive Adaptive Reweighted Sampling; CF: Cascade Forest; CNN: Convolutional Neural Network; COE: Constant Offset Elimination; CR: Continuum Removal; DBSCAN: Density-Based Spatial Clustering of Application with Noise; DF: Deep Forest; DLFM: Deep Learning Feature Fusion Model; DON: deoxynivalenol; FA: factor analysis; Faster R-CNN: Faster Region-based Convolutional Neural Network; FHB: Fusarium head blight; GA: Genetic Algorithm; GP-LVM: Gaussian Process Latent Variable Model; HSI: Hyperspectral imaging; IF: Isolated Forest; ISSPA: Iterative Selection of Successive Projections Algorithm; IVSO: Iterative Variable Subset Optimization; KNN: K-Nearest Neighbors; KPCA: Kernel PCA; KS: Kennard-Stone; LDA: Linear Discriminant Analysis; LDA-QDC: Linear Discriminant Analysis-Quadratic Discriminant Analysis; LS-SVM: Least Square-Support Vector Machine; LWPLSR: Locally Weighted Partial Least Squares Regression; MC: Mean Centering; MD: Mahalanobis distance; MLR: Multiple Linear Regression; MMN: Min–Max Normalization; MSC: Multiplicative Scatter Correction; NIR: Near Infrared; OSC: Orthogonal Signal Correction; PCA: Principal Component Analysis; PLS-DA: Partial Least Squares Discriminant Analysis; PLSR: Partial Least Squares Regression; QPC: Quantification based on pixel counting by classification; RF: Random Frog; SEC: Standard Error of Calibration; SEL: Stacked Ensemble Learning; SEP: Standard Error of Prediction; SFLA: Shuffled Frog Leaping Algorithm; SG: Savitzky–Golay Filtering; SLR: Stepwise Linear Regression; SNV: Standard Normal Variate; SPA: Successive Projections Algorithm; SSAE-CS-SVM: stacked sparse autoencoder combined with a cuckoo search optimized support vector machine; SVM: Support Vector Machine; SVN: Standard Normal Variate; SVR: Support Vector Regression; UVE: Uninformative Variable Elimination; Vis-NIR: Visible and Near-Infrared; VN: vector normalization; WT: Wavelet Transformation; WT-ALSTM: Wavelet Transformation-Attention Mechanism Long Short-term Memory.

## References

[1] An D., Zhang L., Liu Z., Liu J., Wei Y. (2023). Advances in Infrared Spectroscopy and Hyperspectral Imaging Combined with Artificial Intelligence for the Detection of Cereals Quality. Crit. Rev. Food Sci. Nutr..

[2] Priya T.S.R., Manickavasagan A. (2021). Characterising Corn Grain Using Infrared Imaging and Spectroscopic Techniques: A Review. J. Food Meas. Charact..

[3] Zhu M., Huang D., Hu X.-J., Tong W.-H., Han B.-L., Tian J.-P., Luo H.-B. (2020). Application of Hyperspectral Technology in Detection of Agricultural Products and Food: A Review. Food Sci. Nutr..

[4] Mishra G., Panda B.K., Ramirez W.A., Jung H., Singh C.B., Lee S.-H., Lee I. (2021). Research Advancements in Optical Imaging and Spectroscopic Techniques for Nondestructive Detection of Mold Infection and Mycotoxins in Cereal Grains and Nuts. Compr. Rev. Food Sci. Food Saf..

[5] Zhou X., Zhao C., Sun J., Cao Y., Yao K., Xu M. (2023). A Deep Learning Method for Predicting Lead Content in Oilseed Rape Leaves Using Fluorescence Hyperspectral Imaging. Food Chem..

[6] Sun J., Nirere A., Dusabe K.D., Zhong Y., Adrien G. (2024). Rapid and nondestructive watermelon (*Citrullus lanatus*) seed viability detection based on visible near-infrared hyperspectral imaging technology and machine learning algorithms. J. Food Sci..

[7] Yao K., Sun J., Chen C., Xu M., Zhou X., Cao Y., Tian Y. (2022). Non-destructive detection of egg qualities based on hyperspectral imaging. J. Food Eng..

[8] Yao K., Sun J., Cheng J., Xu M., Chen C., Zhou X., Dai C. (2022). Development of simplified models for non-destructive hyperspectral imaging monitoring of S-ovalbumin content in eggs during storage. Foods.

[9] Tian X., Fang Q., Zhang X., Yu S., Dai C., Huang X. (2024). Visualization of moisture content, reducing sugars, and chewiness in bread during oral processing based on hyperspectral imaging technology. Foods.

[10] Yang C., Zhao Y., An T., Liu Z., Jiang Y., Li Y., Dong C. (2021). Quantitative prediction and visualization of key physical and chemical components in black tea fermentation using hyperspectral imaging. LWT.

[11] Sun J., Cao Y., Zhou X., Wu M., Sun Y., Hu Y. (2021). Detection for lead pollution level of lettuce leaves based on deep belief network combined with hyperspectral image technology. J. Food Saf..

[12] Tang N., Sun J., Yao K., Zhou X., Tian Y., Cao Y., Nirere A. (2021). Identification of *Lycium barbarum* varieties based on hyperspectral imaging technique and competitive adaptive reweighted sampling-whale optimization algorithm-support vector machine. J. Food Process. Eng..

[13] Yang F., Sun J., Cheng J., Fu L., Wang S., Xu M. (2023). Detection of starch in minced chicken meat based on hyperspectral imaging technique and transfer learning. J. Food Process. Eng..

[14] Aheto J.H., Huang X., Tian X., Ren Y., Bonah E., Alenyorege E.A., Lv R., Dai C. (2019). Combination of spectra and image information of hyperspectral imaging data for fast prediction of lipid oxidation attributes in pork meat. J. Food Process. Eng..

[15] Xu M., Sun J., Zhou X., Tang N., Shen J., Wu X. (2021). Research on nondestructive identification of grape varieties based on EEMD-DWT and hyperspectral image. J. Food Sci..

[16] Antequera T., Caballero D., Grassi S., Uttaro B., Perez-Palacios T. (2021). Evaluation of fresh meat quality by hyperspectral imaging (HSI), nuclear magnetic resonance (NMR) and magnetic resonance imaging (MRI): A review. Meat Sci..

[17] Ahmad H., Sun J., Nirere A., Shaheen N., Zhou X., Yao K. (2021). Classification of tea varieties based on fluorescence hyperspectral image technology and ABC-SVM algorithm. J. Food Process. Preserv..

[18] Cheng J., Sun J., Yao K., Dai C. (2023). Generalized and hetero two-dimensional correlation analysis of hyperspectral imaging combined with three-dimensional convolutional neural network for evaluating lipid oxidation in pork. Food Control.

[19] Li L., Xie S., Ning J., Chen Q., Zhang Z. (2019). Evaluating green tea quality based on multisensor data fusion combining hyperspectral imaging and olfactory visualization systems. J. Sci. Food Agric..

[20] Yan L., Liu C., Zain M., Cheng M., Huo Z., Sun C. (2024). Estimation of rice protein content based on unmanned aerial vehicle hyperspectral imaging. Agronomy.

[21] Yao K., Sun J., Zhou X., Nirere A., Tian Y., Wu X. (2020). Nondestructive detection for egg freshness grade based on hyperspectral imaging technology. J. Food Process. Eng..

[22] Cheng J., Sun J., Xu M., Zhou X. (2023). Nondestructive detection of lipid oxidation in frozen pork using hyperspectral imaging technology. J. Food Compos. Anal..

[23] Aheto J.H., Huang X., Tian X., Lv R., Dai C., Bonah E., Chang X. (2020). Evaluation of lipid oxidation and volatile compounds of traditional dry-cured pork belly: The hyperspectral imaging and multi-gas-sensory approaches. J. Food Process. Eng..

[24] Xu M., Sun J., Yao K., Wu X., Shen J., Cao Y., Zhou X. (2022). Nondestructive detection of total soluble solids in grapes using VMD-RC and hyperspectral imaging. J. Food Sci..

[25] Li W., Shi Y., Huang X., Li Z., Zhang X., Zou X., Hu X., Shi J., Tomovic V. (2024). Study on the diffusion and optimization of sucrose in Gaido seak based on finite element analysis and hyperspectral imaging technology. Foods.

[26] Ding Y., Zeng R., Jiang H., Guan X., Jiang Q., Song Z. (2024). Classification of tea quality grades based on hyperspectral imaging spatial information and optimization models. J. Food Meas. Charact..

[27] Min D., Zhao J., Bodner G., Ali M., Li F., Zhang X., Rewald B. (2023). Early decay detection in fruit by hyperspectral imaging–Principles and application potential. Food Control.

[28] Wang B., Sun J., Xia L., Liu J., Wang Z., Li P., Guo Y., Sun X. (2023). The applications of hyperspectral imaging technology for agricultural products quality analysis: A review. Food Rev. Int..

[29] Cheng J., Sun J., Shi L., Dai C. (2024). An effective method fusing electronic nose and fluorescence hyperspectral imaging for the detection of pork freshness. Food Biosci..

[30] Delwiche S.R., Rodriguez I.T., Rausch S.R., Graybosch R.A. (2019). Estimating percentages of fusarium-damaged kernels in hard wheat by near-infrared hyperspectral imaging. J. Cereal Sci..

[31] Song Y., Cao S., Chu X., Zhou Y., Xu Y., Sun T., Zhou G., Liu X. (2023). Non-destructive detection of moisture and fatty acid content in rice using hyperspectral imaging and chemometrics. J. Food Compos. Anal..

[32] Ma J., Sun D.-W., Pu H., Cheng J.-H., Wei Q. (2019). Advanced techniques for hyperspectral imaging in the food industry: Principles and recent applications. Annu. Rev. Food Sci. Technol..

[33] Tian Y., Sun J., Zhou X., Wu X., Lu B., Dai C. (2020). Research on apple origin classification based on variable iterative space shrinkage approach with stepwise regression-support vector machine algorithm and visible-near infrared hyperspectral imaging. J. Food Process. Eng..

[34] Femenias A., Gatius F., Ramos A.J., Teixido-Orries I., Marin S. (2022). Hyperspectral imaging for the classification of individual cereal kernels according to fungal and mycotoxins contamination: A review. Food Res. Int..

[35] Dai C., Sun J., Huang X., Zhang X., Tian X., Wang W., Sun J., Luan Y. (2023). Application of hyperspectral imaging as a nondestructive technology for identifying tomato maturity and quantitatively predicting lycopene content. Foods.

[36] Tian Y., Sun J., Zhou X., Yao K., Tang N. (2022). Detection of soluble solid content in apples based on hyperspectral technology combined with deep learning algorithm. J. Food Process. Preserv..

[37] Shi Y., Wang Y., Hu X., Li Z., Huang X., Liang J., Zhang X., Zhang D., Zou X., Shi J. (2023). Quantitative characterization of the diffusion behavior of sucrose in marinated beef by HSI and FEA. Meat Sci..

[38] Xu F., Huang X., Tian X., Yu S., Zhang X., Zareef M. (2024). Application of hyperspectral imaging and colorimetric sensor array coupled with multivariate analysis for quality detection during salted duck eggs processing. J. Food Process. Eng..

[39] Shao Y., Gao C., Xuan G., Gao X., Chen Y., Hu Z. (2020). Determination of damaged wheat kernels with hyperspectral imaging analysis. Int. J. Agric. Biol. Eng..

[40] Wu X., Liang X., Wang Y., Wu B., Sun J. (2022). Non-destructive techniques for the analysis and evaluation of meat quality and safety: A review. Foods.

[41] Tian X.-Y., Aheto J.H., Dai C., Ren Y., Bai J.-W. (2021). Monitoring microstructural changes and moisture distribution of dry-cured pork: A combined confocal laser scanning microscopy and hyperspectral imaging study. J. Sci. Food Agric..

[42] Lu B., Sun J., Yang N., Hang Y. (2019). Fluorescence hyperspectral image technique coupled with HSI method to predict solanine content of potatoes. J. Food Process. Preserv..

[43] Aheto J.H., Huang X., Tian X., Bonah E., Ren Y., Alenyorege E.A., Dai C. (2019). Investigation into crystal size effect on sodium chloride uptake and water activity of pork meat using hyperspectral imaging. J. Food Process. Preserv..

[44] Shi Y., Wang Y., Hu X., Li Z., Huang X., Liang J., Zhang X., Zheng K., Zou X., Shi J. (2023). Nondestructive discrimination of analogous density foreign matter inside soy protein meat semi-finished products based on transmission hyperspectral imaging. Food Chem..

[45] Shi L., Sun J., Zhang B., Wu Z., Jia Y., Yao K., Zhou X. (2024). Simultaneous detection for storage condition and storage time of yellow peach under different storage conditions using hyperspectral imaging with multi-target characteristic selection and multi-task model. J. Food Compos. Anal..

[46] Nirere A., Sun J., Atindana V.A., Hussain A., Zhou X., Yao K. (2022). A comparative analysis of hybrid SVM and LS-SVM classification algorithms to identify dried wolfberry fruits quality based on hyperspectral imaging technology. J. Food Process. Preserv..

[47] Saha D., Manickavasagan A. (2021). Machine learning techniques for analysis of hyperspectral images to determine quality of food products: A review. Curr. Res. Food Sci..

[48] Tang N., Jun S., Min X., Yao K., Yan C., Liu D. (2022). Identification of fumigated and dyed *Lycium barbarum* by hyperspectral imaging technology. J. Food Process. Eng..

[49] He P., Wu Y., Wang J., Ren Y., Ahmad W., Liu R., Ouyang Q., Jiang H., Chen Q. (2020). Detection of mites *Tyrophagus putrescentiae* and *Cheyletus eruditus* in flour using hyperspectral imaging system coupled with chemometrics. J. Food Process. Eng..

[50] Zhu L., Ma Q., Chen J., Zhao G. (2022). Current progress on innovative pest detection techniques for stored cereal grains and thereof powders. Food Chem..

[51] Zhang X., Wang Y., Zhou Z., Zhang Y., Wang X. (2023). Detection method for tomato leaf mildew based on hyperspectral fusion terahertz technology. Foods.

[52] Xing F., Yao H., Liu Y., Dai X., Brown R.L., Bhatnagar D. (2019). Recent developments and applications of hyperspectral imaging for rapid detection of mycotoxins and mycotoxigenic fungi in food products. Crit. Rev. Food Sci. Nutr..

[53] Cao Y., Li H., Sun J., Zhou X., Yao K., Nirere A. (2020). Nondestructive determination of the total mold colony count in green tea by hyperspectral imaging technology. J. Food Process. Eng..

[54] Li J., Luo W., Han L., Cai Z., Guo Z. (2022). Two-wavelength image detection of early decayed oranges by coupling spectral classification with image processing. J. Food Compos. Anal..

[55] Williams P.J., Bezuidenhout C., Rose L.J. (2019). Differentiation of maize ear rot pathogens, on growth media, with near infrared hyperspectral imaging. Food Anal. Methods.

[56] Lv Y., Lv W., Han K., Tao W., Zheng L., Weng S., Huang L. (2022). Determination of wheat kernels damaged by *Fusarium* head blight using monochromatic images of effective wavelengths from hyperspectral imaging coupled with an architecture self-search deep network. Food Control.

[57] Liang K., Ren Z., Song J., Yuan R., Zhang Q. (2024). Wheat FHB resistance assessment using hyperspectral feature band image fusion and deep learning. Int. J. Agric. Biol. Eng..

[58] Femenias A., Llorens-Serentill E., Ramos A.J., Sanchis V., Marin S. (2022). Near-infrared hyperspectral imaging evaluation of *Fusarium* damage and DON in single wheat kernels. Food Control.

[59] Chakraborty S.K., Mahanti N.K., Mansuri S.M., Tripathi M.K., Kotwaliwale N., Jayas D.S. (2021). Non-destructive classification and prediction of aflatoxin-B1 concentration in maize kernels using Vis-NIR (400–1000 nm) hyperspectral imaging. J. Food Sci. Technol..

[60] Mansuri S.M., Chakraborty S.K., Mahanti N.K., Pandiselvam R. (2022). Effect of germ orientation during Vis-NIR hyperspectral imaging for the detection of fungal contamination in maize kernel using PLS-DA, ANN and 1D-CNN modelling. Food Control.

[61] Teixido-Orries I., Molino F., Gatius F., Sanchis V., Marin S. (2023). Near-infrared hyperspectral imaging as a novel approach for T-2 and HT-2 toxins estimation in oat samples. Food Control.

[62] Su W.-H., Yang C., Dong Y., Johnson R., Page R., Szinyei T., Hirsch C.D., Steffenson B.J. (2021). Hyperspectral imaging and improved feature variable selection for automated determination of deoxynivalenol in various genetic lines of barley kernels for resistance screening. Food Chem..

[63] Nie S., Gao W., Liu S., Li M., Li T., Ren J., Ren S., Wang J. (2024). Hyperspectral imaging combined with deep learning models for the prediction of geographical origin and fungal contamination in millet. Front. Sustain. Food Syst..

[64] Wang Z., Fu Z., Weng W., Yang D., Wang J. (2022). An efficient method for the rapid detection of industrial paraffin contamination levels in rice based on hyperspectral imaging. LWT.

[65] Femenias A., Bainotti M.B., Gatius F., Ramos A.J., Marin S. (2021). Standardization of near infrared hyperspectral imaging for wheat single kernel sorting according to deoxynivalenol level. Food Res. Int..

[66] Kurmi Y., Saxena P., Kirar B.S., Gangwar S., Chaurasia V., Goel A. (2022). Deep CNN model for crops’ diseases detection using leaf images. Multidimens. Syst. Signal Process..

[67] Xue B., Tian L., Wang Z., Wang X., Yao X., Zhu Y., Cao W., Cheng T. (2023). Quantification of rice spikelet rot disease severity at organ scale with proximal imaging spectroscopy. Precis. Agric..

[68] Zhang F., Cui X., Zhang C., Cao W., Wang X., Fu S., Teng S. (2022). Rapid non-destructive identification of selenium-enriched millet based on hyperspectral imaging technology. Czech J. Food Sci..

[69] Wang S., Sun J., Fu L., Xu M., Tang N., Cao Y., Yao K., Jing J. (2022). Identification of red jujube varieties based on hyperspectral imaging technology combined with CARS-IRIV and SSA-SVM. J. Food Process. Eng..

[70] Liang J., Wang Y., Shi Y., Huang X., Li Z., Zhang X., Zou X., Shi J. (2023). Non-destructive discrimination of homochromatic foreign materials in cut tobacco based on VIS-NIR hyperspectral imaging. J. Sci. Food Agric..

[71] Zhang L., Sun J., Zhou X., Nirere A., Wu X., Dai R. (2020). Classification detection of saccharin jujube based on hyperspectral imaging technology. J. Food Process. Preserv..

[72] Fu L., Sun J., Wang S., Xu M., Yao K., Cao Y., Tang N. (2022). Identification of maize seed varieties based on stacked sparse autoencoder and near-infrared hyperspectral imaging technology. J. Food Process. Eng..

[73] Bai Z., Tian J., Hu X., Sun T., Luo H., Huang D. (2022). A back-propagation neural network model using hyperspectral imaging applied to variety nondestructive detection of cereal. J. Food Process. Eng..

[74] Sivakumar C., Chaudhry M.M.A., Paliwal J. (2022). Classification of pulse flours using near-infrared hyperspectral imaging. LWT.

[75] Lei Y., Hu X., Tian J., Zhang J., Yan S., Xue Q., Ma X., Chen M., Huang D. (2022). Rapid resolution of types and proportions of broken grains using hyperspectral imaging and optimization algorithm. J. Cereal Sci..

[76] Ekramirad N., Doyle L., Loeb J., Santra D., Adedeji A.A. (2024). Hyperspectral imaging and machine learning as a nondestructive method for proso millet seed detection and classification. Foods.

[77] Zhu D., Han J., Liu C., Zhang J., Qi Y. (2025). Vis-NIR and NIR hyperspectral imaging combined with convolutional neural network with attention module for flaxseed varieties identification. J. Food Compos. Anal..

[78] Bu Y., Jiang X., Tian J., Hu X., Han L., Huang D., Luo H. (2023). Rapid nondestructive detecting of sorghum varieties based on hyperspectral imaging and convolutional neural network. J. Sci. Food Agric..

[79] Han L., Tian J., Huang Y., He K., Liang Y., Hu X., Xie L., Yang H., Huang D. (2024). Hyperspectral imaging combined with dual-channel deep learning feature fusion model for fast and non-destructive recognition of brew wheat varieties. J. Food Compos. Anal..

[80] Ozdogan G., Gowen A. (2025). Wheat grain classification using hyperspectral imaging: Concatenating Vis-NIR and SWIR data for single and bulk grains. Food Control.

[81] Zhu J., Li H., Rao Z., Ji H. (2023). Identification of slightly sprouted wheat kernels using hyperspectral imaging technology and different deep convolutional neural networks. Food Control.

[82] Jiang X., Bu Y., Han L., Tian J., Hu X., Zhang X., Huang D., Luo H. (2023). Rapid nondestructive detecting of wheat varieties and mixing ratio by combining hyperspectral imaging and ensemble learning. Food Control.

[83] Shao Y., Xuan G., Hu Z., Wang Y. (2019). Detection of adulterants and authenticity discrimination for coarse grain flours using NIR hyperspectral imaging. J. Food Process. Eng..

[84] Bai Z., Hu X., Tian J., Chen P., Luo H., Huang D. (2020). Rapid and nondestructive detection of sorghum adulteration using optimization algorithms and hyperspectral imaging. Food Chem..

[85] Huang H., Hu X., Tian J., Peng X., Luo H., Huang D., Zheng J., Wang H. (2022). Rapid and nondestructive determination of sorghum purity combined with deep forest and near-infrared hyperspectral imaging. Food Chem..

[86] Zhang L., Sun H., Rao Z., Ji H. (2020). Non-destructive identification of slightly sprouted wheat kernels using hyperspectral data on both sides of wheat kernels. Biosyst. Eng..

[87] Huang H., Liu Y., Zhu S., Feng C., Zhang S., Shi L., Sun T., Liu C. (2024). Detection of mechanical damage in corn seeds using hyperspectral imaging and the ResNeSt_E deep learning network. Agriculture.

[88] Kadam S., Pabrekar S., Sawardekar S., Barage S. (2023). High-throughput and molecular interventions for identification and characterization of rice germplasm. Cereal Res. Commun..

[89] Nirere A., Sun J., Kama R., Atindana V.A., Nikubwimana F.D., Dusabe K.D., Zhong Y. (2023). Nondestructive detection of adulterated wolfberry (*Lycium Chinense*) fruits based on hyperspectral imaging technology. J. Food Process. Eng..

[90] Sun J., Yang F., Cheng J., Wang S., Fu L. (2024). Nondestructive identification of soybean protein in minced chicken meat based on hyperspectral imaging and VGG16-SVM. J. Food Compos. Anal..

[91] Alvarez J., Martinez E., Diezma B. (2021). Application of hyperspectral imaging in the assessment of drought and salt stress in magneto-primed triticale seeds. Plants.

[92] Tian X.-Y., Aheto J.H., Bai J.-W., Dai C., Ren Y., Chang X. (2021). Quantitative analysis and visualization of moisture and anthocyanins content in purple sweet potato by Vis-NIR hyperspectral imaging. J. Food Process. Preserv..

[93] Cheng J., Sun J., Yao K., Xu M., Tian Y., Dai C. (2022). A decision fusion method based on hyperspectral imaging and electronic nose techniques for moisture content prediction in frozen-thawed pork. LWT.

[94] Zhang L., An D., Wei Y., Liu J., Wu J. (2022). Prediction of oil content in single maize kernel based on hyperspectral imaging and attention convolution neural network. Food Chem..

[95] Mendoza P.T.D., Armstrong P.R., Peiris K.H.S., Siliveru K., Bean S.R., Pordesimo L.O. (2023). Prediction of sorghum oil content using near-infrared hyperspectral imaging. Cereal Chem..

[96] Zhang Y., Guo W. (2020). Moisture content detection of maize seed based on visible/near-infrared and near-infrared hyperspectral imaging technology. Int. J. Food Sci. Technol..

[97] Zhang Y., Yang T., Wang Z., Li S., Chen Y. (2024). In situ detection of moisture content and gelatinization degree during rice processing using hyperspectral imaging. J. Food Compos. Anal..

[98] Han L., Jiang X., Zhou S., Tian J., Hu X., Huang D., Luo H. (2024). Hyperspectral imaging technology combined with the extreme gradient boosting algorithm (XGBoost) for the rapid analysis of the moisture and acidity contents in fermented grains. J. Am. Soc. Brew. Chem..

[99] Huang Y., Tian J., Yang H., Hu X., Han L., Fei X., He K., Liang Y., Xie L., Huang D. (2024). Detection of wheat saccharification power and protein content using stacked models integrated with hyperspectral imaging. J. Sci. Food Agric..

[100] Fei X., Jiang X., Lei Y., Tian J., Hu X., Bu Y., Huang D., Luo H. (2023). The rapid non-destructive detection of the protein and fat contents of sorghum based on hyperspectral imaging. Food Anal. Methods.

[101] He K., Tian J., Hu X., Fei X., Han L., Huang Y., Liang Y., Xie L., Yang H., Huang D. (2024). Rapid and non-destructive determination of the protein and fat contents in wheat by hyperspectral imaging combined with AdaBoost-SVR modeling. J. Am. Soc. Brew. Chem..

[102] Jiang X., Tian J., Huang H., Hu X., Han L., Huang D., Luo H. (2022). Nondestructive visualization and quantification of total acid and reducing sugar contents in fermented grains by combining spectral and color data through hyperspectral imaging. Food Chem..

[103] Zhang J., Lei Y., He L., Hu X., Tian J., Chen M., Huang D., Luo H. (2023). The rapid detection of the tannin content of grains based on hyperspectral imaging technology and chemometrics. J. Food Compos. Anal..

[104] Hu N., Li W., Du C., Zhang Z., Gao Y., Sun Z., Yang L., Yu K., Zhang Y., Wang Z. (2021). Predicting micronutrients of wheat using hyperspectral imaging. Food Chem..

[105] Shi T., Gao Y., Song J., Ao M., Hu X., Yang W., Chen W., Liu Y., Feng H. (2024). Using VIS-NIR hyperspectral imaging and deep learning for non-destructive high-throughput quantification and visualization of nutrients in wheat grains. Food Chem..

[106] Huang H., Hu X., Tian J., Jiang X., Sun T., Luo H., Huang D. (2021). Rapid and nondestructive prediction of amylose and amylopectin contents in sorghum based on hyperspectral imaging. Food Chem..

[107] Liang Y., Tian J., Hu X., Huang Y., He K., Xie L., Yang H., Huang D., Zhou Y., Xia Y. (2024). Rapid determination of starch and alcohol contents in fermented grains by hyperspectral imaging combined with data fusion techniques. J. Food Sci..

[108] Blanch-Perez-del-Notario C., Saeys W., Lambrechts A. (2020). Fast ingredient quantification in multigrain flour mixes using hyperspectral imaging. Food Control.

[109] Zhong Y., Sun J., Yao K., Cheng J., Du X. (2024). Detection of rice (with husk) moisture content based on hyperspectral imaging technology combined with MSLPP-ESMA-SVR model. J. Food Saf..

[110] Qiao M., Xu Y., Xia G., Su Y., Lu B., Gao X., Fan H. (2022). Determination of hardness for maize kernels based on hyperspectral imaging. Food Chem..

[111] Cheng J., Sun J., Yao K., Xu M., Zhou X. (2022). Nondestructive detection and visualization of protein oxidation degree of frozen-thawed pork using fluorescence hyperspectral imaging. Meat Sci..

[112] Medina-Garcia M., Roca-Nasser E.A., Martinez-Domingo M.A., Valero E.M., Arroyo-Cerezo A., Cuadros-Rodriguez L., Jimenez-Carvelo A.M. (2024). Towards the establishment of a green and sustainable analytical methodology for hyperspectral imaging-based authentication of wholemeal bread. Food Control.

[113] Kang Z., Zhao Y., Chen L., Guo Y., Mu Q., Wang S. (2022). Advances in machine learning and hyperspectral imaging in the food supply chain. Food Eng. Rev..

[114] Bian H., Ma B., Yu G., Dong F., Li Y., Xu Y., Tan H. (2024). Fusion features of microfluorescence hyperspectral imaging for qualitative detection of pesticide residues in Hami melon. Food Res. Int..

[115] ElMasry G., Gou P., Al-Rejaie S. (2021). Effectiveness of specularity removal from hyperspectral images on the quality of spectral signatures of food products. J. Food Eng..

[116] Peng J., Zhang J., Han L., Ma X., Hu X., Lin T., He L., Yi X., Tian J., Chen M. (2024). Determination of malathion content in sorghum grains using hyperspectral imaging technology combined with stacked machine learning models. J. Food Compos. Anal..

[117] Hu X., Zhang J., Lei Y., Tian J., Peng J., Chen M. (2024). Classification of pesticide residues in sorghum based on hyperspectral and gradient boosting decision trees. J. Food Saf..

[118] Quinn B., McCarron P., Hong Y., Birse N., Wu D., Elliott C.T., Ch R. (2022). Elementomics combined with DD-SIMCA and K-NN to identify the geographical origin of rice samples from China, India, and Vietnam. Food Chem..

[119] Choi J.-Y., Kim H.-C., Moon K.-D. (2021). Geographical origin discriminant analysis of chia seeds (*Salvia hispanica* L.) using hyperspectral imaging. J. Food Compos. Anal..

[120] Edris M., Ghasemi-Varnamkhasti M., Kiani S., Yazdanpanah H., Izadi Z. (2024). Identifying the authenticity and geographical origin of rice by analyzing hyperspectral images using unsupervised clustering algorithms. J. Food Compos. Anal..

[121] Van De Steene J., Ruyssinck J., Fernandez-Pierna J.-A., Vandermeersch L., Maes A., Van Langenhove H., Walgraeve C., Demeestere K., De Meulenaer B., Jacxsens L. (2023). Fingerprinting methods for origin and variety assessment of rice: Development, validation and data fusion experiments. Food Control.

